# One Hundred and Sixty-three Cases of Fracture of the Neck of the Femur

**Published:** 1879-01

**Authors:** H. Wardner

**Affiliations:** Cairo, Ills., Chairman of the Committee on Surgery, Southern Illinois Medical Association


### ARTICLE I.

### ONE HUNDRED AND SIXTY-THREE CASES OF FRACTURE OF THE NECK OF THE FEMUR. COLLECTED AND REPORTED BY H. WARDNER, M.D., CAIRO, ILLS., Chairman of the Committee on Surgery, Southern Illinois Medical Association.

Fracture of the neck of the femur is a subject of special interest to all surgeons who have had cases under treatment. Having had my attention called to this subject, I addressed a circular to quite a large number of the older practitioners, asking for reports of such cases as may have come under their observation.

In response I received reports, more or less in detail, of 163 cases which are embodied in the following tabulated statement. One hundred and three of these cases are reported as having occurred within the capsule, sixteen as being both intra and extra capsular, thirty-six as extra capsular, one as a separation of the epiphysis, and seven as uncertain.

| Name of Surgeon. | No. of Patient. | Sex. | Age when it Occurred. | Date of Occurrence. | Character of Fracture. | Right or Left Side. | Extra or Intra-Capsular. | TREATMENT. What application or dressing was used? When first seen by Surgeon, Etc., Etc. | RESULT. Union or Non-Union, Ligamentous or Bony. Amount of Shortening. Degree of Usefulness. |
|---|---|---|---|---|---|---|---|---|---|
| P. A. Allaire | 1 | F. | 97 | 1873. | Simple. | R. | Intra. | No appliance used. | Got up very well, and lived to 101 years. |
| P. A. Allaire | 2 | F. | 86 | 1875. | Simple. | L. | Intra. | No appliance used. | Died of exhaustion in 8 days. |
| P. A. Allaire | 3 | F. | 75 | 1876. | Simple. | L. | Intra. | No appliance used. | Died of exhaustion in 30 days. |
| A. T. Bartlett | 4 | F. | 57 | Dec. 2d, 1867. | Simple, obscure. | R. | Intra. | Seen soon after accident. Hodgen's splint. | Ligamentous union. 1.2 Cm. shortening. |
| S. C. Plummer | 5 | F. | 65 | Feb. 20th, 1875. | Simple. Transverse. | R. | Intra. | 15 hours after injury. Truesdale's fracture-bed. | Bony union. No shortening. |
| J. H. Reeder | 6 | F. | 58 | 1872. | Simple. | R. | Intra. | 4 to 6 weeks in double inclined plane with extension by weight from knee, followed by reclining chair, and no restraint. | Union ligamentous *probably*. Limb quite useful. 5 Cm. shortening. |
| H. Culbertson | 7 | F. | 73 | July 5th, 1858. | Simple. | L. | Intra. | Simply supported on pillows. | Non-union. New joint formed. Used crutch. Died in 1866, of senile gangrene. Shortening 3.7 Cm. |
| S. C. Plummer | 8 | F. | 77 | Feb. 5th, 1871. | Simple. | R. | Intra. | Truesdale's fracture-bed. | Non-union. Died in 21 days of old age. |
| S. C. Plummer | 9 | F. | 78 | Aug. 15th 1873. | Simple. Transverse. Oblique. | R. | Intra. | Truesdale's fracture-bed. | Perfect union in 42 days, but patient died of old age and confinement in treatment. |
| H. Wardner | 10 | M. | 65 | January, 1859. | Simple. | R. | Intra. | Seen soon after injury. McIntyre's splint, and double inclined plane. | Union probably ligamentous. Walked with cane after two years. Shortening not determined. |
| H. Wardner | 11 | F. | 69 | March 14 1872. | Simple. | R. | Intra. | Seen at once. Extension by weight and pulley. | Non-union, or possibly ligamentous. Uses crutch. About 5 Cm. shortening. |
| H. Wardner | 12 | M. | 16 | 1876. | Simple. | R. | Intra. | Hodgen's splint, and plaster Paris. | Bony union. Walks with cane. Shortening but little, and not determined. |
| L. Dyer | 13 | F. | 60 | | Simple. | — | Intra. | Physick's modified splint. | Non or ligamentous union. Walks with crutch. 5 Cm. short. |
| P. A. Allaire | 14 | F. | 62 | 1874. | Simple. | R. | Intra. | No appliance used. | Limb useful with a cane. |

| Name of Surgeon. | No. of Patient. | Sex. | Age when it Occurred. | Date of Occurrence. | Character of Fracture. | Right or Left Side. | Extra or Intra-Capsular. | TREATMENT. What application or dressing was used? When first seen by Surgeon, etc., etc. | RESULT. Union or Non-Union, Ligamentous or Bony. Amount of Shortening. Degree of usefulness, |
|---|---|---|---|---|---|---|---|---|---|
| A. E. Bell | 15 | F. | 62 | Jan. 29th, 1867. | Simple. | R. | Intra, with free laceration of ligament. | No splints. Simple dressings. | Non-union. False joint. 3.1 Cm. shortening. |
| D. A. K. Steele | 16 | F. | | April 22d, 1876. | Simple. | L. | Intra. | Seen night following the injury. Buck's apparatus with weight and pulley. | Non-union. Eversion Limb nearly useless. Spindle-celled sarcoma removed from over tensor vaginæ femoris 18 months after. About 2.5 Cm. short. |
| M. M. Goodman | 17 | F. | 80 | 1871. | Simple. | R. | Intra. | No dressings except liniments. | Non-union. Limb about useless. But little shortening. |
| Saml. H. Birney | 18 | F. | 56 | July 8th, 1872. | Simple. Transverse. | R. | Intra. | Seen 3 hours after injury. Double inclined plane of Daniel's fracture-bed. | Union bony. Good use of limb. 2.5 Cm. short. |
| L. Dyer | 19 | M. | 50 | | Simple. | — | Intra. | Physick's splint modified. Extension and counter-extension. | Union ligamentous *probably*. Walks with crutch. 5 Cm. short. |
| L. H. Stroud | 20 | F. | 50 | Oct. 1876. | Simple. | L. | Intra. | Supposed to have been a contusion. No dressing used. | Non-union. But little use. |
| G. Callen | 21 | F. | 30 | 1865. | Simple. | R. | Intra. | Bathed with camphor ! | Supposed to be ligamentous. Helpless without crutches. 5 Cm. short. |
| Chas. Richards | 22 | F. | 84 | 1866. | Simple. Transverse. | R. | Intra. | Seen 4 hours after injury. Long splint and bandage. | Union ligamentous probably. Walked with crutches. Shortening not measured. |
| Chas. Richards | 23 | F. | 61 | 1870. | Simple. Transverse. | L. | Intra. | Seen 2 hours after injury. Long splint and bandage. | Union ligamentous (?) No use of limb. 7.6 Cm. short. |
| Chas. Richards, and J. Casey | 24 | F. | 58 | 1877. | Simple. Transverse. | L. | Intra. | Seen 2 hours after injury. Long splint and bandage. | Had no use of limb to time of death, 3 months later. 5 Cm. shortening. |
| M. A. McClelland | 25 | F. | 60 | 1871. | Simple. Transverse. | R. | Intra (probably). | Was first seen by a "doctor" who pronounced it a sprain. No dressing whatever. | Union ligamentous. Walks with crutch. Shortening not determined. |
| J. H. Reeder | 26 | F. | 65 | 1870. | Simple. Transverse. | R. | Intra. | Double-inclined plane 4 to 6 with weight extension from knee. Afterward reclining chair and no restraint. | Union ligamentous, probably. Limb useful. 3.7 Cm. short. |
| C. H. Mastin | 27 | M. | 64 | 1875. | Simple. | L. | Intra. | Seen at once. Amesbury's double inclined plane. | Union ligamentous. Walks very well. |

| Name of Surgeon. | No. of Patient. | Sex. | Age when it Occurred. | Date of Occurrence. | Character of Fracture. | Right or Left Side. | Extra or Intra-Capsular. | TREATMENT. What application or dressing was used? When first seen by Surgeon, etc., etc. | RESULT. Union or Non-union, Ligamentous or Bony. Amount of Shortening. Degree of Usefulness. |
|---|---|---|---|---|---|---|---|---|---|
| W. Meacher ............ | 28 | M. | 70 | 1872. | Simple. | R. | Intra. | Placed on pillows. | Non-union (probably ligamentous). Could walk with cane. |
| W. Meacher............. | 29 | F. | 60 | 1871. | Simple. | R. | Intra. | Placed on pillows. | Non-union. Could walk with cane. 1.2 Cm. shortening. |
| W. Meacher ............ | 30 | F. | 25 | 1875. | Simple. | L. | Intra. | Extension by weight and pulley. | Union ligamentous. Walks with cane. 1.2 Cm. shortening. |
| D. B. Trimble........... | 31 | M. | 80 | 1858. | Simple. | L. | Intra. | Caused by lifting. Lived 5 months. | Non-union. |
| D. B. Trimble........... | 32 | M. | 75 to 80 | 1860. | Simple. | R. | Intra. | Jumped from second story window. Lived 6 weeks. | Non-union. |
| D. B. Trimble........... | 33 | F. | 90 | 1863. | Simple. | — | Intra. | Caused by pulling open a bureau drawer. | Died in 2 days. |
| E. R. Willard .......... | 34 | M. | 35 | Jan. 5th, 1874. | Supposed impacted. | L. | Intra. | Long splint. | Union probably ligamentous. Usefulness good. 3.7 Cm. short. |
| E. R. Willard........... | 35 | F. | 32 | Sept. 28, 1874. | Simple. | L. | Intra. | Long splint. | Union probably ligamentous. Good use. 1.2 Cm. short. |
| Chas. T. Parkes........ | 36 | F. | 72 | October, 1874. | Simple. Oblique. | R. | Intra, probably. | Seen after 4 hours. Double inclined plane 2 weeks. Confinement not insisted on. | Union ligamentous. Walks with crutch and cane. 3.7 Cm. short. |
| J. H. Ledlie ............ | 37 | M. | 74 | April, 1868. | Simple. | L. | Intra. | No dressing. | Died in a few days. Never rallied from shock. |
| A. G. Williams......... | 38 | M. | 61 | Aug. 1st, 1871. | Simple. | L. | Intra. | Seen soon after accident. Long splint and extension. | Union ligamentous. Limb nearly useless. 2.4 Cm. short. Eversion. |
| R. G Bogue ............ | 39 | F. | 90 | Mch. 7th, 1876. | Simple. | L. | Intra. | Limb flexed and laid over padded blanket under knee. | Died of exhaustion April 2d, 1876. |
| R. G. Bogue ............ | 40 | F. | 92 | Dec. 4th, 1876. | Simple. | L. | Intra. | Limb flexed with a pillow under knee. | Died of exhaustion Jan. 2, 1877. |
| F. L. Wadsworth ... . | 41 | F. | 75 | N'v. 30th. 1877. | Simple. | R. | Intra. | Limb flexed with a pillow under knee. | Non-union. Uses crutches since the sixth week. |
| Henry Tomboken..... | 42 | F. | 82 | Jan. 10th, 1878. | Simple. | R. | Intra. | Same treatment as above. | Non-union. Not useful; but not painful. |
| R. W. Crothers......... | 43 | M. | 80 | Febru'ry, 1876. | Simple. | L. | Intra. | Same as above. | Non-union Limb useless. Died the following year. |

| Name of Surgeon. | No. of Patient. | Sex. | Age when it Occurred. | Date of Occurrence. | Character of Fracture. | Right or Left Side. | Extra or Intra-Capsular. | TREATMENT. What application or dressing was used? When first seen by surgeon. etc., etc. | RESULT. Union or Non-Union, Ligamentous or Bony. Amount of Shortening. Degree of usefulness. |
|---|---|---|---|---|---|---|---|---|---|
| R. L. Moore ............ | 44 | F. | 81 | Sept. 15, 1874. | Comminuted. | L. | Intra. | Seen 4 days after injury. Extension by weight and pulley, and double inclined plane. | Death on 18th day from exhaustion. |
| R. L. Moore............ | 45 | F. | 69 | Oct. 15, 1874. | Simple. | L. | Intra. | Seen ten days after occurrence. Extension by weight and pulley, and sole leather splint. | Union ligamentous. Always used crutches. Shortening not known. |
| R. L Moore............ | 46 | F. | 73 | June 5, 1875. | Impacted. | R. | Intra. | Seen soon after accident. Double inclined plane with weight and pulley. | Died on 54th day, from septicæmia. Autopsy verified diagnosis. |
| M. L. Hall, reported by G. W. Jones...... | 47 | F. | 76 | Feb. 28, 1877. | Impacted. | R. | Intra. | No treatment allowed by patient. | Ligamentous union. Some usefulness. 3 Cm. short. |
| A. C. Rankin............ | 48 | M. | 81 | Jan. 1st, 1874. | Simple. | R. | Intra. | Long straight splint was used 8 weeks. | Non-union. Limb useless and painful. Died at end of one year. |
| Wm. T. Kirk........... | 49 | F. | 63 | March 5, 1873. | Simple. | L. | Intra. | Seen early. On 3d day placed limb in fracture box with side splints extending to axilla and perineum, with extension. Kept in bed three months. | Union bony. Walked without crutch or cane after 18 months. 2.4 Cm. short. |
| J. H. Ledlie ............ | 50 | F. | 58 | Nov., 1858. | Simple. | L. | Intra. | Seen immediately. Double inclined plane and pillows. | Union ligamentous. Used crutches. 7.6 Cm. short. |
| R. Boal and J. C. Frye | 51 | M. | 30 | Dec. 12, 1868. | Simple. | L. | Intra. | No dressings. | Probably bony. Walks with cane. 3.1 Cm. short. |
| Wm. M. Chambers... | 52 | F. | 71 | 1855. | Simple. | L. | Intra. | Sir Astley Cooper's method. No dressings. | Non-union. Walked with cane or crutch. 5 Cm. short. |
| Wm. M. Chambers... | 53 | F. | 69½ | Feb. 23, 1878. | Simple. | L. | Intra. | No dressings. | Non-union. Walking with crutches. Shortening unknown. |
| No Surgeon.............. | 54 | F. | 71 | 1873. | Simple. | L. | Intra. | No treatment. | Non-union. 5 Cm. short. |
| H. W. Kendall......... | 55 | F. | 69 | Sept., 1866. | Simple. | R. | Intra. | Cause, tripped on carpet. Weight and pulley, 3 months in recumbent position. [See note.] | Limb quite useful. Walks with cane. Union Ligamentous. 6.3 Cm. shortening. |
| H. W. Kendall......... | 56 | F. | 43 | August, 1877. | Simple. | R. | Intra. | Weight, pulley and long splint. | Unable to walk without crutches. Non-union. 2.5 Cm. short. |

| Name of Surgeon. | No. of Patient. | Sex. | Age when it Occurred. | Date of Occurrence. | Character of Fracture. | Right or Left Side. | Extra or Intra-Capsular. | TREATMENT. What application or dressing was used? When first seen by Surgeon, etc., etc. | RESULT. Union or Non-union, Ligamentous or Bony. Amount of Shortening, Degree of Usefulness. |
|---|---|---|---|---|---|---|---|---|---|
| S. L. Cheaney | 57 | F. | 98 | Sept. 2, 1870. | Simple. | R. | Intra. (?) | Sand bags and rest. | Was able to get about with a ligamentous union. |
| Wm. B. Alley | 58 | M. | 78 | June, 1861. | Simple. | L. | Intra. | Seen after a few days. No dressings. | Union ligamentous. Walked only on crutches. 5 Cm. short. |
| Wm. B. Alley | 59 | F. | 82 | Sept., 1863. | Simple. | L. | Intra. | Seen at once. Hagedorn's (?) apparatus. | Union ligamentous. Used crutches 7 years before death. 5 Cm. shortening. |
| Wm. B. Alley | 60 | M. | 75 | Dec., 1871. | Impacted. | R. | Intra. | Same as above. | Union supposed to be ligamentous. 6.3 Cm. short. |
| Theodore Meyer | 61 | F. | 45 | May 4, 1849. | Simple. | R. | Intra. | Seen early. Spica bandage with starch and glue. | Probably union (ligamentous?). Used crutches last heard from; but doing well. |
| C. K. Hendee | 62 | F. | 59 | Sept., 1869. | Simple. | R. | Intra. | Seen 10 days after injury. Splint with extension and counter-extension. | Union ligamentous. Can walk with cane. 3.7 Cm. short. |
| C. K. Hendee | 63 | F. | 68 | Feb., 1875. | Simple. | R. | Intra. | Seen 3 months after injury. No dressing. | Union ligamentous. Uses crutch. 7.6 Cm. short. |
| C. K. Hendee | 64 | M. | 62 | Jan'y, 1877. | Simple. | R. | Intra. | No dressing except straw bolsters to keep leg from twisting. | Union ligamentous. Walks with cane. 5 Cm. short. |
| R. Roskoten | 65 | F. | 70 | 1868. | Simple. | R. | Intra. | Seen after 8 weeks. Was first treated for dislocation by attending physician. No dressing when seen by me. | Non-union. |
| Kingkade, rep'ted by Dr. D. O. Morrell | 66 | M. | 75 | Sept. 14, 1877. | Simple. | R. | Intra. | Seen 6 weeks after injury. Hodgen's extension splint and plast. Paris. | Non-union. |
| A. S. Munsell, rep'ted by F. A. Emmons | 67 | F. | 80 | Jan. 25th, 1875. | Simple. | R. | Intra. | Seen 2 weeks after injury. Extension. | Non-union. Cannot move limb. 1.8 Cm. short. |
| Chas. T. Parkes | 68 | F. | 76 | Sept., 1877. | Simple. | L. | Intra. | Seen within 24 hours. No special dressings. Arranged on pillows. | Non-union probably. Can move limb. No pain. Bed ridden from old age. |
| R. W. Crothers | 69 | F. | 60 | Jan'y, 1859. | Simple. | R. | Intra. | Seen in 3 hours. Bandage, extension and wet paste board from knee to crest of ilium. | Ligamentous union. Walks with cane or crutch. Shortening not perceptible. |
| R. W. Crothers | 70 | F. | 63 | Jan'y, 1866. | Simple. | L. | Intra. | Seen within half an hour. Dressing same as above. | Union ligamentous. Walks with crutch or cane. Shorten'g not obsrvd. |

| Name of Surgeon. | No. of Patient. | Sex. | Age when it Occurred. | Date of Occurrence. | Character of Fracture. | Right or Left Side. | Extra or Intra-Capsular. | TREATMENT. What application or dressing was used? When first seen by surgeon, etc., etc. | RESULT. Union or Non-Union, Ligamentous or Bony. Amount of shortening. Degree of Usefulness. |
|---|---|---|---|---|---|---|---|---|---|
| I. N. Danforth......... | 71 | M. | 70 | 1865. | Simple. | R. | Intra. | Patient was a physician, and a very impatient patient. Starch dressing only. | Osseous union. Walks with cane. 2.4 Cm. shortening. |
| I. N. Danforth......... | 72 | F. | 60 | 1864. | Simple. | R. | Intra. | Elevation foot of bed. Sand-bags, weight and pulley. | Osseous union. Walks with a little limp. 1.8 Cm. shortening. |
| C. H. Mastin............ | 73 | F. | 8 | ——— | Simple. | R. | Intra. Supposed separation of Epiphysis. | Seen several months after union. Kape's apparatus for fracture of hip. | Walks well with splint. 2.7 Cm. shortening. |
| C. H. Mastin............ | 74 | F. | 60 | Dec., 1877. | Simple. | L. | Intra. | Inclined plane and weight for several weeks. Rest in bed. | Ligamentous union. Walks with crutches. |
| C. H. Mastin............ | 75 | F. | 58 | July, 1876 | Simple. | L. | Intra. | 15 months after occurrence applied Agnew's splint for coxalgia, with perineal band. | Cannot walk without apparatus. Does very well with it. 2.4 Cm. shortening. |
| Wm. B. Alley........... | 76 | F. | 76 | Sept. 1876 | Simple. | L. | Intra. | No dressing. | Died in 10 days of pneumonia. |
| J. L. Miller, reported by J. S. Whitmire.. | 77 | F. | 50 | Dec., 1852. | Simple. | —— | Intra. | Suppose simplest kind, as the surgeon was too penurious to procure proper apparatus. | No union whatever. Never walked without crutches. 10 Cm. shortening. |
| E. L. Marshall......... | 78 | F. | 62 | October, 1857. | Simple. | R. | Intra. | Seen 4th day. Daniel's fracture-bed. | Union ligamentous (?) Nearly useless. 3.7 Cm. short. |
| E. L. Marshall......... | 79 | F. | 61 | Aug. 30, 1875. | Impacted. | R. | Intra. | Rest, and sand bags. | Ligamentous perhaps. Walks with ease with cane. Slight shortening. |
| D. S. Booth.............. | 80 | F. | 55 | 1865. | Simple. | L. | Intra. | Foot of bed raised 2½ inches. Extension by weights. Sand bag support. | Union Ligamentous. Amount of shortening undetermined. |
| D. S. Booth.............. | 81 | F. | 60 | 1872. | Simple. | L. | Intra. | Same as above. | Same as above. |
| A. J. Mead............... | 82 | F. | 68 | 1863. | Simple. | R. | Intra. | Seen 3 months after accident. No dressings. | Union Ligamentous. But little use. 3.7 Cm. short. |
| M. J. Roeschlaub...... | 83 | M. | 42 | June 19, 1851. | Simple. | R. | Intra? | Double inclined plane after Dupuytren and Richards. Seen within an hour. | Union bony. Useful. 0.6 Cm. short. |
| S. L. Cheaney.......... | 84 | F. | 56 | Aug. 1st, 1867. | Simple. | R. | Intra? | Long spilnt with heavy weights. | Union bony. Quite useful after six months. Shortening undetermined. |

| Name of Surgeon. | No. of Patient. | Sex. | Age when it Occurred. | Date of Occurrence. | Character of Fracture. | Right or Left Side. | Extra or Intra-Capsular. | TREATMENT. What application or dressing was used? When first seen by Surgeon, etc., etc. | RESULT. Union or Non-Union, Ligamentous or Bony. Amount of Shortening. Degree of Usefulness. |
|---|---|---|---|---|---|---|---|---|---|
| Allin & Sams | 85 | F. | 55 | Sept., 1855. | Simple. | L. | Intra. | Extension at first, but discontinued 3d day, too painful. | Union ligamentous probably. Limb useless. 1.8 Cm. short, and 4 years after 3.7 Cm. short. |
| John Schmidt | 86 | M. | 62 | 1874. | Simple. | L. | Intra. | No dressing. Recumbent posture. | Limb quite useful with raised heel. 3.7 Cm. short. |
| W. H. H. King | 87 | F. | 61 | July, 1874. | Simple. | L. | Intra. | No dressing. Recumbent position. | Union ligamentous. Walks with crutch and cane. |
| E. Gaylord | 88 | F. | 67 | Jan. 25, 1870. | Impacted. | L. | Intra. | No dressing. Recumbency. | Union bony. Slight eversion. 2.4 Cm. short. |
| E. Gaylord | 89 | F. | 84 | Oct. 31, 1874. | Impacted. | L. | Intra. | No dressing. Recumbency. | Union bony. Some lameness. 3.7 inches short. |
| Thomas Whitten | 90 | F. | 74 | Dec. 20, 1876. | Simple. | L. | Intra. | Seen one week after accident. Extension with long splint. | Union ligamentous. Usefulness much impaired. 3.7 Cm. short. |
| W. J. Chenoweth | 91 | M. | 70 | Nov., 1873. | Simple. | R. | Intra. | Long splint and roller. | Non-union. Patient delirious and would not endure dressings. |
| H. C. Howard | 92 | F. | 52 | ——— | Simple. | R. | Intra. | Extension and rest. | No union. |
| Chas. C. F. Gay | 93 | F. | 60 | 1871. | Simple. | L. | Intra. | Fracture and shortening, only recognized after some weeks, after tripping on the carpet. No dressings used. | Non-union. Shortening 6.3 Cm. |
| Chas. C. F. Gay | 94 | M. | 52 | 1875. | Simple. | L. | Intra. | Extension. | Non-union. Shortening 1.8 Cm. Subsequently died of cancer of stomach. |
| Chas. C. F. Gay | 95 | M. | 67 | March 5, 1870. | Simple. | L. | Intra. | Extension by weight and pulley. | Non-union. 1.8 Cm. shortening. |
| Chas. C. F. Gay | 96 | M. | 32 | June 8, 1870. | Simple. | L. | Intra. | Extension by weight and pulley. | Non-union. Shortening 2.5 Cm. |
| Chas. C. F. Gay | 97 | M. | 61 | 1875. | Simple. | L. | Intra. | Fell striking on hip. No dressings. | Non-union. 5 Cm. shortening. |
| Chas. C. F. Gay | 98 | M. | 64 | October, 1876. | Simple. | R. | Intra. | Large man, fell striking on left hip. No dressings. | Non-union. 4.7 Cm. short. Died 9 months after injury. |
| H. T. Montgomery | 99 | F. | 70 | 1871. | Simple. | L. | Intra. | Foot of bed 9 inches high. 12 to 16 pounds over pulleys, two parallel. | Union. Walked in 18 months. 1.8 Cm. short. No splint used. |

| Name of Surgeon. | No. of Patient. | Sex. | Age When it Occurred. | Date of Occurrence. | Character of Fracture. | Right or Left Side. | Extra or Intra-Capsular. | TREATMENT. What application or dressing was used? When first seen by Surgeon, etc., etc. | RESULT. Union or Non-union, Ligamentous or Bony. Amount of Shortening. Degree of Usefulness. |
|---|---|---|---|---|---|---|---|---|---|
| Abner Hard............ | 100 | M. | 70 | 1855. | Simple. Transverse. | R. | Intra. | Seen immediately after injury. First used long splint, but soon changed to pulley and weight and continued to recovery. | Ligamentous union probably. Little shortening. Patient first went on crutches, but finally with cane. Useful leg. |
| Abner Hard. .......... | 101 | F. | 63 | 1858. | Simple. Transverse. | R. | Intra. | Seen soon after fracture occurred. Treated by pulley and weight till recovery. | Suppose ligamentous union. Little shortening. After first year was able to go without crutch or cane. Useful limb. |
| Abner Hard............ | 102 | F. | 64 | Dec. 1, 1866. | Simple. Transverse. | R. | Intra. | Seen soon after injury. Treated by pulley and weight. | Suppose ligamentous union. Little shortening. Went first with crutch and then with cane. Useful limb. |
| Abner Hard............ | 103 | F. | 78 | Sept. 14, 1877. | Simple. Transverse. | R. | Intra. | Seen soon after injury. Pulley and weight and subsequently by starch bandage. | Do not know as to union. Patient is using crutches and improving steadily. Little shortening. |
| H. Wardner............ | 104 | F. | 56 | 1877. | Simple. | R. | Intra and perhaps partly Extra | Hodgen's splint and plaster Paris. | Union seems to be bony. Walks now with crutch. 0.6 Cm. short. A little eversion. |
| E. L. Marshall......... | 105 | F. | 65 | July 5, 1860. | Simple. Oblique. | L. | Intra and Extra. | Seen seventh day. Daniel's fracture bed. | Probably bony. Walked with cane. 5 Cm. short. |
| Chas. T. Parkes........ | 106 | F. | 45 | Nov., 1876. | Simple. Oblique. | R. | Intra and Extra. | Seen within 24 hours. Extension by weight and pulley and long external splint. | Good bony union. Useful limb. 2.5 Cm. or more short. |
| Chas. T. Parkes....... | 107 | M. | 42 | Febru'ry, 1878. | Impacted. | R. | Intra and Extra. | Weight and pulley and sand bags. | Bony union. Good limb; *some limp.* 1.8 Cm. short. |
| Chas. T. Parkes, Cook Co. Hospital......... | 108 | M. | 40 | 1877. | Simple. Oblique. | L. | Intra and Extra. | Weight and pulley. | Bony union. Good limb. 0.6 Cm. short. |
| Chas. T. Parkes........ | 109 | M. | 65 | March, 1875. | Simple. Oblique. | L. | Intra and Extra. | Seen within 24 hours. Weight and pulley and long external splint. | Union bony. Useful but lame. 2.1 Cm. short. |
| S. C. Plummer and G. G. Craig............ | 110 | M. | 49 | March, 1871. | Simple. Oblique. | R. | Extra and Intra. | 24 hours after injury. Truesdale's fracture bed. | Bony union. Perfect use. Longer than opposite limb. |
| I. N. Danforth......... | 111 | F. | 68 | 1875. | Simple. | L. | Oblique, Extra and Intra. | Weight and pulley extension. Blankets and splints. | Union not certain; believed to be bony. Walks well but complains of weakness. 2.5 Cm. shortening. |

| Name of Surgeon. | No of Patient. | Sex. | Age When it Occurred. | Date of Occurrence. | Character of Fracture. | Right or Left Side. | Extra or Intra-Capsular. | TREATMENT. What application or dressing was used? When first seen by Surgeon, etc., etc. | RESULT. Union or Non-Union, Ligamentous or Bony. Amount of Shortening. Degree of Usefulness. |
|---|---|---|---|---|---|---|---|---|---|
| F. M. Agnew............ | 112 | F. | 70 | May, 1869. | Simple. | R. | Extra and perhaps Intra. | Straight splint, perfect rest. | Union bony. Walks very well with a cane. |
| C Richards............... | 113 | M. | 22 | 1874. | Simple. Oblique. | R. | Extra and Intra. | Sole leather splint and weight for extension. | Bony union. Motion impaired. Fair use. No shortening. |
| R. P. Lane, reported by G. W. Rohr...... | 114 | F. | 54 | 1877. | Simple. Oblique. | R. | Extra and Intra. | Weight and pulley. | Union bony. 3.1 Cm. short. |
| Various Army Surgeons.................. | 115 | M. | 40 | July 19, 1864. | Compound. | R. | Extra and Intra. | Seen at once. Laid on well side. Cooling lotions. No splints. See note. | Bony union. Usefulness impaired. 3.1 Cm. short. |
| H. C. Howard........... | 116 | F. | 58 | ——— | Simple. | L. | Extra and Intra. | Extension, immobility, rest. | Good recovery. 1.8 Cm. short. |
| H. C. Howard.......... | 117 | F. | 45 | ——— | Impacted. | R. | Not certain. | Splints, immobility. No extension. | 3.7 short. |
| Joseph Teft.............. | 118 | M. | 75 | 1855. | Oblique. | R. | Extra and Intra. | No treatment. | Non-union. |
| D. Prince.................. | 119 | M. | 35 | April 8, 1875. | Simple, Impacted. | R. | Extra and Intra. | Hodgen's splint. Seen 20 days after injury. | Death, 25 weeks after injury, from drinking liniment containing sulphuric acid, while in delirium tremens. |
| Drs. Howell, reported by Abner Hard..... | 120 | M. | 68 | 1876. | Simple. Transverse | R. | Extra. | No dressing. Fracture was not detected for several months; supposed to be a contusion. | Non-union. Shortening 6.3 Cm. No use of limb. Moves by use of crutches. |
| D. A. K. Steele......... | 121 | M. | —— | April 25, 1877. | Impacted. | L. | Extra. | Seen 3 days after injury. Hodgen's splint. | Bony union. Very useful limb. No lameness. 0.6 Cm. short. |
| Saml. H. Birney....... | 122 | M. | 64 | Jan. 12, 1874. | Comminuted. | R. | Extra. | 6 hours after fracture. Recumbency, extension by weight and pulley. | Bony union. Eversion. Walks well with cane. 3.7 Cm. short. |
| Saml. H. Birney....... | 123 | M. | 3 | Jan. 2, 1878. | Comminuted. | R. | Extra. | Seen 5 days after injury. Double, straight thigh splints. Extension by weight and pulley. | Bony union. Began to walk at end of 5 weeks. Not over 1.2 Cm. short. |
| Z. C. McElroy.......... | 124 | F. | 55 | 1855. | Simple. Obscure. | L. | Extra. | No apparatus. Placed on pillows. | No union by bone. Walked on crutches 15 years, and died of pneumonia. 5 Cm. short. |
| J. Stonemetz............ | 125 | M. | 12 | 1862. | Simple. Transverse. | L. | Extra. | Liston's splint. | Bony union. Perfect use. No perceptible shortening. |

| Name of Surgeon. | No. of Patient. | Sex. | Age When it Occurred. | Date of Occurrence | Character of Fracture. | Right or Left Side. | Extra or Intra-Capsular. | TREATMENT. What application or dressing was used? When first seen by Surgeon, etc., etc. | RESULT. Union or Non-Union, Ligamentous or Bony. Amount of Shortening. Degree of Usefulness. |
|---|---|---|---|---|---|---|---|---|---|
| Chas. Reber............ | 126 | F. | 65 | 1872. | Simple. | L. | Extra. | Seen 24 hours after accident. Extension by weight and pulley. | Union ligamentous. Can walk with cane or crutch. 2.5 Cm. shortening. |
| W. Meacher........... | 127 | M. | 60 | 1873. | Simple. | L. | Extra. | Sand bags. Extension by weight and pulley. | Good recovery. Walks well. 0.6 Cm. short. |
| J. R. Wells............. | 128 | F. | 60 | Dec. 19, 1874. | Simple. | L. | Extra. | Truesdale's fracture bed. | Union bony. Perfect use. No perceptible shortening. |
| H. Culbertson......... | 129 | M. | 27 | July 10, 1854. | Simple, with dislocation of ankle same side, and simple fracture of both bones of left leg. | R. | Extra. Note. | Double inclined plane. Extension could not be borne. Supported by pillows. | Union bony. Limb useful. 6.3 Cm. short. |
| R. Roskoten............ | 130 | M. | 51 | Jan. 14, 1858. | Simple, with contusions. | L. | Extra. | Dzondis' apparatus, improved by Hagedorn, for extension, and the splint of Burminghasen to support fracture in the proper position. | Bony union. Good use of limb. 1.8 Cm. short. |
| Fred. Brendel.......... | 131 | M. | 44 | Nov. 22, 1869. | Simple. | R. | Extra. | Straight splint and extension. | Union bony. Tolerably good use of leg, which was shortened by a previous fracture. Total shortening 3.7 Cm. |
| A. J. Mead.............. | 132 | F. | 70 | 1856. | Simple. | L. | Extra. | Seen at end of 4 months. No dressings. | Union ligamentous. Never walked without crutch. 5 Cm. shortening. |
| J. Stout.................. | 133 | F. | 70 | March 19, 1869. | Simple. | R. | Extra. | Extension by weight. | Union bony. Limb useful. 1.8 Cm. shortening. |
| C. B. Reed ............. | 134 | M. | 70 | Sept. 28, 1866. | Simple. | R. | Extra. | Seen 3 hours after injury. Limb supported by cushions. | Ligamentous union. Usefulness much impaired. 5 Cm. shortening. |
| Chas. Reber............ | 135 | F. | 63 | 1869. | Simple. | R. | Extra. | Seen in 6 hours. Extension by weight and pulley. | Ligamentous union. Walks with cane or crutch. 2.5 Cm. shortening. |
| W. J. Chenoweth...... | 136 | F. | 19 | Aug. 28, 1877. | Impacted. | R. | Extra. | Long splint and roller. | Good union. No appreciable shortening. |
| W. J. Chochran........ | 137 | M. | 52 | January, 1876. | Impacted. | L. | Extra. | No displacement. Long splint and rest. | Bony union. Slight eversion of foot. 1.2 Cm. short. Walks with cane. |
| J. H. Ledlie............ | 138 | M. | 20 | April 10, 1862. | Compound, G. S. W. | L. | Extra. | Seen one month after accident. Extension with weight and pulley. | Union bony; joint anchylosed. Good use. 3.7 Cm. short. |

| Name of Surgeon. | No. of Patient. | Sex. | Age When it Occurred. | Date of Occurrence. | Character of Fracture. | Right or Left Side. | Extra or Intra-Capsular. | TREATMENT. What application or dressing was used? When first seen by Surgeon, etc., etc. | RESULT. Union or Non-Union, Ligamentous or Bony. Amount of Shortening, Degree of Usefulness. |
|---|---|---|---|---|---|---|---|---|---|
| T. M. Sams | 139 | F. | 36 | Dec., 1849. | Impacted. | R. | Extra. | Long splint and extension. | Good union; bony. Useful but imperfect. 1.2 Cm. short. |
| F. B. Haller | 140 | M. | 70 | 1852. | Simple. | R. | Extra. Anatomical neck. (?) | Seen 4 hours after injury. Straight splint. | *Died* 3rd day from internal injuries. |
| C. H. Richings, reported by G. W. Rhor. | 141 | F. | 55 | 1872. | Simple. Transverse. | L. | Extra. | Long straight splint. | Union bony. No perceptible shortening. |
| B. K. Lucas | 142 | M. | 71 | Jan. 9, 1877. | Simple. | R. | Extra. | Extension by weight and pulley at once. | Bony union. Can walk long distance. 1.2 Cm. short. |
| Josiah Whitnel | 143 | M. | 43 | 1858. | Simple. | L. | Extra. | Seen first day. Used Day's splint. | Bony union. 1.2 Cm. short. |
| Josiah Whitnel | 144 | M. | 70 | 1866. | Simple. | R. | Extra. | Seen 2 months after injury. No dressings used. | Union ligamentous. 7.5 Cm. short. |
| Josiah Whitnel | 145 | M. | 69 | 1866. | Simple. | R. | Extra. | Seen first day. Used Day's splint. | Non-union. |
| Wm. M. Chambers and A. Evans | 146 | M. | 40 | 1850. | Simple. | L. | Extra. | Liston's splint. | Non-union (ligamentous?). Walked with cane. 3.7 Cm. short. |
| Wm. H. H. King | 147 | F. | 58 | August 2, 1870. | Simple. | R. | Extra. | Seen in a few hours. Dressed with weight and pulley. | Union bony. 1.8 Cm. short. |
| Drs. Monroe and Speed | 148 | M. | 35 | May 29, 1872. | Simple. Transverse. | R. | Extra. | Seen at once. Dressed in Day's splint (?) with extension and counter-extension. | Union supposed to be bony. Patient *died* in six months of phthisis. Shortening 1.2 Cm. |
| Chas. C. F. Gay | 149 | M. | 27 | May, 1874. | Simple and comp'nd fracture of tibia | L. | Extra. | Fell from a boat. Extension simply. | Non-union. 1.8 Cm. short. |
| Chas. C. F. Gay | 150 | M. | 55 | October, 1874. | Simple. | L. | Extra. | Extension. | Bony union. 5 Cm. short. Walks with cane and high heel. |
| H. F. Montgomery | 151 | F. | 25 | — | Simple. | L. | Extra. | Long splint with screw extension. | United. 2.2 Cm. short. |
| Chas. C. F. Gay | 152 | F. | 24 | 1877. | Simple. | R. | Cervix, part not stated. | No statement. | Non-union. Shortening 5 Cm. Toes everted. Limb much atrophied. |
| J. F. Stewart | 153 | F. | 3 | April 22, 1871. | Simple. | R. | Extra. | Seen at once. Applied simple straight splint. | Bony union. No perceptible shortening. Perfect use. |
| J. F. Stewart | 154 | M. | 56 | Feb. 17, 1873. | Simple. | R. | Extra. | Seen at once. Extension by weight and pulley. | Bony union. Almost perfect use. 1.2 Cm. short. |
| J. F. Stewart and J. Murphy | 155 | M. | 64 | July 12, 1875. | Simple. | R. | Extra. | Seen at once. Straight splint and extension. | Bony union. Usefulness much impaired, but improving. 3.1 Cm. short. |

| Name of Surgeon. | No. of Patient. | Sex. | Age When it Occurred. | Date of Occurrence. | Character of Fracture. | Right or Left Side. | Extra or Intra-Capsular. | TREATMENT. What application or dressing was used? When first seen by Surgeon, etc., etc. | RESULT. Union or Non-Union, Ligamentous or Bony. Amount of Shortening. Degree of Usefulness. |
|---|---|---|---|---|---|---|---|---|---|
| Drs. Monroe and Speed.................. | 156 | M. | 9 | June 18, 1876. | Simple. Transverse. | L. | Probably a separation of epiphysis. | Seen soon after accident. Physick's splint and junk bags. | Union bony. No apparent shortening. Usefulness perfect. |
| Edmund Andrews..... | 157 | M. | Ab't 60 | Forgotten. | Simple. | R. | I do not know any way to make a decisive diagnosis on this point. Many cases are doubtless partly intra and partly extra capsular. I suppose those who got a bony union were probably *at least partly* extra capsular. | Side splint, adhesive straps, pulley and weight. | Apparently bony union. Able to walk well with cane. |
| Edmund Andrews..... | 158 | M. | Ab't 40 | Forgotten. | Simple. | R. | | Adhesive straps, pulley and weight. | Bony union. Good use. |
| Edmund Andrews..... | 159 | F. | 36 | Forgotten. | Impacted. | Forgotten. | | Horizontal position only. | Bony union. Good use. |
| Edmund Andrews..... | 160 | F. | Ab't 60 | ——— | Impacted. | L. | | Horizontal position only. | Union doubtful. Imperfect use. |
| Edmund Andrews..... | 161 | M. | 45 | ——— | Simple. | Forgotten. | | Adhesive straps, pulley and weight. | Bony union. Good use. |
| Edmund Andrews..... | 162 | M. | Ab't 35 | ——— | Compound. | R. | | Splints. | Bony union. Considerable use. |
| Edmund Andrews..... | 163 | M. | Ab't 38 | ——— | Simple. | Forgotten. | | Forgotten. | No union. Imperfect use. |

DEAR DOCTOR: My records are imperfect on this subject. I send such as I am able to recall with distinctness. I distrust the precision of all attempts to diagnosticate extra from intra-capsular. In cases of old persons who fail of union, I generally presume the fracture is intra-capsular, but I doubt the certainty of the conclusion, even when corroborated by the other symptoms usually relied on to help out the inference.

The following table shows amount of shortening resulting from the different modes of treatment as reported:

| MODE OF TREATMENT. | Total number of cases. | No. of cases in which the amount of shortening is given. | Maximum. Cm. | Minimum. Cm. | Average. | REMARKS. |
|---|---|---|---|---|---|---|
| No appliance used. Support on pillows.. | 48 | 21 | 10. | 3.1 | 5. | Non-union 8, ligamentous 11, bony 21. |
| Extension by weight and pulley........... | 42 | 34 | 6.3 | .0 | 2.1 | |
| Hodgen's modification of Smith's ant.... | 6 | 4 | 1.2 | 0.6 | 1. | |
| Truesdale's fracture bed...................... | 5 | 4 | .0 | .0 | | One longer than natural. |
| Daniels' fracture bed.......................... | 3 | 3 | 5. | 2.5 | 3.7 | |
| Amesbury's double inclined plane......... | 7 | 5 | 7.6 | 0.6 | 4. | |
| Straight splint, with extension after the manner of Physick.......................... | 27 | 18 | 7.6 | .0 | 2. | |
| Other apparatus of seven different kinds, but involving same principles as above | 25 | 9 | 6.3 | 1.2 | 2.8 | |

Many eminent surgeons distrust accuracy of diagnosis in a majority of cases of this injury. The difficulty of correct diagnosis is very well illustrated in the case of Bergman vs. Volker, Superior court of Buffalo, N. Y., September term, 1876.

Plaintiff was sixty-one years old; was knocked into a gutter by a passing team, striking on left side, August 31st, 1875. Was lifted into a wagon and started home; stopped to see a doctor, who said it was only a bruise. At home, cried with pain in left hip when shoe and sock were removed. Four days later, called Dr. Walker, who diagnosticated and treated the injury as a bruise.

Dr. Gay saw the case 18th of May, 1876, made careful examination, and pronounced the case dislocation upon dorsum of ilium, and made what he supposed to be a reduction, but afterwards concluded the head of the bone had slipped into the sciatic notch.

Dr. Miner diagnosticated a fracture of the neck. Dr. T. F. Rochester ditto. Dr. James P. White diagnosticated fracture within capsular ligament. Dr. H. A. Hopkins diagnosticated dislocation into sciatic notch.

On March 22d, 1877, plaintiff committed suicide. A post mortem examination revealed the facts set forth in the following:

"This is to certify, that on the 22d day of March, 1877, we, the undersigned, made a post mortem examination of the left hip of Chas. Bergman, of this city. The head of the femur was in the acetabulum. There had been a fracture of the neck of the femur, followed by its entire absorption. The shaft of the femur was united to the head by a long, strong ligament.

"(Signed), R. W. VAN PEYMA, M. D.,
*Post Mortem Examiner of Erie Co., N. Y.*
BERNARD BARTOW, M. D."

The examination was made in the presence of Drs. White, Miner, Rochester, Gay, Wycoff, Wetmore, Hauenstine, Diehl, Storck, W. W. Miner, Brush, Doggett, Hopkins, Folwell and others.

In regard to the modes of treatment, the results as given are favorable to the use of Hodgen's splint and Truesdale's fracture bed; but the number of cases reported is not sufficient to warrant a decisive opinion. My own experience in the use of the former has been eminently satisfactory. Those who have used the latter are enthusiastic in its praise.

Results are certainly in favor of treatment by appliances intended to retain the fragments in apposition.

I regret that time and the pressure of urgent duties prevent my making the study of these cases that I had intended when the subject was first entertained. I place them upon record, feeling that they will be of use to others whose attention may be drawn to the subject. I desire to acknowledge valuable aid from Dr. H. Y. Stalker, of Cairo, in tabulating the cases.

